# Previous fracture and subsequent fracture risk: A meta-analysis to update FRAX

**DOI:** 10.1007/s00198-023-06870-z

**Published:** 2023-08-11

**Authors:** John A Kanis, Helena Johansson, Eugene V McCloskey, Enwu Liu, Kristina E Åkesson, Fred A Anderson, Rafael Azagra, Cecilie L Bager, Charlotte Beaudart, Heike A Bischoff-Ferrari, Emmanuel Biver, Olivier Bruyère, Jane A Cauley, Jacqueline R Center, Roland Chapurlat, Claus Christiansen, Cyrus Cooper, Carolyn J Crandall, Steven R Cummings, José AP da Silva, Bess Dawson-Hughes, Adolfo Diez-Perez, Alyssa B Dufour, John A Eisman, Petra JM Elders, Serge Ferrari, Yuki Fujita, Saeko Fujiwara, Claus-Christian Glüer, Inbal Goldshtein, David Goltzman, Vilmundur Gudnason, Jill Hall, Didier Hans, Mari Hoff, Rosemary J Hollick, Martijn Huisman, Masayuki Iki, Sophia Ish-Shalom, Graeme Jones, Magnus K Karlsson, Sundeep Khosla, Douglas P Kiel, Woon-Puay Koh, Fjorda Koromani, Mark A Kotowicz, Heikki Kröger, Timothy Kwok, Olivier Lamy, Arnulf Langhammer, Bagher Larijani, Kurt Lippuner, Dan Mellström, Thomas Merlijn, Anna Nordström, Peter Nordström, Terence W O’Neill, Barbara Obermayer-Pietsch, Claes Ohlsson, Eric S Orwoll, Julie A Pasco, Fernando Rivadeneira, Anne Marie Schott, Eric J Shiroma, Kristin Siggeirsdottir, Eleanor M Simonsick, Elisabeth Sornay-Rendu, Reijo Sund, Karin MA Swart, Pawel Szulc, Junko Tamaki, David J Torgerson, Natasja M van Schoor, Tjeerd P van Staa, Joan Vila, Nicholas J Wareham, Nicole C Wright, Noriko Yoshimura, M Carola Zillikens, Marta Zwart, Liesbeth Vandenput, Nicholas C Harvey, Mattias Lorentzon, William D Leslie

**Affiliations:** Mary McKillop Institute for Health Research, Australian Catholic University, Melbourne, Australia; Centre for Metabolic Bone Diseases, University of Sheffield, Sheffield, UK; Mary McKillop Institute for Health Research, Australian Catholic University, Melbourne, Australia; Sahlgrenska Osteoporosis Centre, Institute of Medicine, University of Gothenburg, Sweden; Centre for Metabolic Bone Diseases, University of Sheffield, Sheffield, UK; MRC Versus Arthritis Centre for Integrated research in Musculoskeletal Ageing, Mellanby Centre for Musculoskeletal Research, University of Sheffield, Sheffield, UK; Mary McKillop Institute for Health Research, Australian Catholic University, Melbourne, Australia; Clinical and Molecular Osteoporosis Research Unit, Department of Clinical Sciences, Lund University, Lund, Sweden; Department of Orthopedics, Skåne University Hospital, Malmö, Sweden; GLOW Coordinating Center, Center for Outcomes Research, University of Massachusetts Medical School, Worcester, MA, USA; Department of Medicine, Autonomous University of Barcelona, Barcelona, Spain; Health Centre Badia del Valles, Catalan Institute of Health, Barcelona, Spain; GROIMAP (research group), Unitat de Suport a la Recerca Metropolitana Nord, Institut Universitari d’investigació en Atenció Primària Jordi Gol, Cerdanyola del Vallès, Barcelona, Spain; PRECIOSA-Fundación para la investigación, Barberà del Vallés, Barcelona, Spain; Nordic Bioscience A/S, Herlev, Denmark; WHO Collaborating Centre for Public Health Aspects of Musculoskeletal Health and Aging, Division of Public Health, Epidemiology and Health Economics, University of Liège, Liège, Belgium; Department of Health Services Research, University of Maastricht, Maastricht, the Netherlands; Department of Aging Medicine and Aging Research, University Hospital, Zurich, and University of Zurich, Zurich, Switzerland; Centre on Aging and Mobility, University of Zurich and City Hospital, Zurich, Switzerland; Division of Bone Diseases, Department of Medicine, Geneva University Hospitals and Faculty of Medicine, University of Geneva, Geneva, Switzerland; WHO Collaborating Centre for Public Health Aspects of Musculoskeletal Health and Aging, Division of Public Health, Epidemiology and Health Economics, University of Liège, Liège, Belgium; Department of Epidemiology, School of Public Health, University of Pittsburgh, Pittsburgh, Philadelphia, United States.; Skeletal Diseases Program, Garvan Institute of Medical Research, Sydney, NSW, Australia; St Vincent’s Clinical School, School of Medicine and Health, University of New South Wales Sydney, Sydney, NSW, Australia; School of Medicine Sydney, University of Notre Dame Australia, Sydney, NSW, Australia; INSERM UMR 1033, Université Claude Bernard-Lyon1, Hôpital Edouard Herriot, Lyon, France; Nordic Bioscience A/S, Herlev, Denmark; MRC Lifecourse Epidemiology Centre, University of Southampton, Southampton, UK; NIHR Southampton Biomedical Research Centre, University of Southampton and University Hospitals Southampton NHS Foundation Trust, Southampton, UK; NIHR Oxford Biomedical Research Unit, University of Oxford, Oxford, UK; Division of General Internal Medicine and Health Services Research, David Geffen School of Medicine, University of California, Los Angeles, CA, USA; San Francisco Coordinating Center, California Pacific Medical Center Research Institute, San Francisco, CA, USA; Coimbra Institute for Clinical and Biomedical Research, Faculty of Medicine, University of Coimbra, Coimbra, Portugal; Rheumatology Department, Centro Hospitalar e Universitário de Coimbra, Coimbra, Portugal; Bone Metabolism Laboratory, Jean Mayer US Department of Agriculture Human Nutrition Research Center on Aging, Tufts University, Boston, MA, USA; Department of Internal Medicine, Hospital del Mar and CIBERFES, Autonomous University of Barcelona, Barcelona, Spain; Marcus Institute for Aging Research, Hebrew SeniorLife, Boston, MA, USA; Department of Medicine, Beth Israel Deaconess Medical Center and Harvard Medical School, Boston, MA, USA; Skeletal Diseases Program, Garvan Institute of Medical Research, Sydney, NSW, Australia; St Vincent’s Clinical School, School of Medicine and Health, University of New South Wales Sydney, Sydney, NSW, Australia; School of Medicine Sydney, University of Notre Dame Australia, Sydney, NSW, Australia; Petra JM Elders Department of General Practice, Amsterdam UMC, location AMC, Amsterdam Public Health Research Institute, Amsterdam, The Netherlands; Division of Bone Diseases, Department of Medicine, Geneva University Hospitals and Faculty of Medicine, University of Geneva, Geneva, Switzerland; Center for Medical Education and Clinical Training, Kindai University Faculty of Medicine, Osaka, Japan; Department of Pharmacy, Yasuda Women’s University, Hiroshima, Japan; Section Biomedical Imaging, Molecular Imaging North Competence Center, Department of Radiology and Neuroradiology, University Medical Center Schleswig-Holstein Kiel, Kiel University, Kiel, Germany; Maccabitech Institute of Research and Innovation, Maccabi Healthcare Services, Tel Aviv, Israel; Department of Epidemiology and Preventive Medicine, School of Public Health, Sackler Faculty of Medicine, Tel Aviv University, Tel Aviv, Israel; Department of Medicine, McGill University and McGill University Health Centre, Montreal, Canada; Icelandic Heart Association, Kopavogur, Iceland; University of Iceland, Reykjavik, Iceland; MRC Centre for Reproductive Health, University of Edinburgh, Edinburgh, UK; Interdisciplinary Centre of Bone Diseases, Bone and Joint Department, Lausanne University Hospital (CHUV) & University of Lausanne, Lausanne, Switzerland; Department of Neuromedicine and Movement Science, Norwegian University of Science and Technology, Trondheim, Norway; Department of Rheumatology, St Olavs Hospital, Trondheim, Norway; Aberdeen Centre for Arthritis and Musculoskeletal Health, Epidemiology Group, University of Aberdeen, Aberdeen, UK; Department of Epidemiology and Data Science, Amsterdam Public Health Research Institute, VU University Medical Center, Amsterdam, The Netherlands; Department of Sociology, VU University, Amsterdam, The Netherlands; Department of Public Health, Kindai University Faculty of Medicine, Osaka, Japan; Endocrine Clinic, Elisha Hospital, Haifa, Israel; Menzies Institute for Medical Research, University of Tasmania, Hobart, Australia; Clinical and Molecular Osteoporosis Research Unit, Department of Clinical Sciences, Lund University, Lund, Sweden; Department of Orthopaedics, Skåne University Hospital, Malmö, Sweden; Robert and Arlene Kogod Center on Aging and Division of Endocrinology, Mayo Clinic College of Medicine, Mayo Clinic, Rochester, MN, USA; Marcus Institute for Aging Research, Hebrew Senior Life, Boston, MA, USA; Department of Medicine, Beth Israel Deaconess Medical Center and Harvard Medical School, Boston, MA, USA; Healthy Longevity Translational Research Programme, Yong Loo Lin School of Medicine, National University of Singapore; Singapore Institute for Clinical Sciences, Agency for Science Technology and Research (A*STAR), Singapore; Department of Internal Medicine, Erasmus University Medical Center, Rotterdam, The Netherlands; Department of Radiology and Nuclear Medicine, Erasmus University Medical Center, Rotterdam, The Netherlands; Deakin University, IMPACT (Institute for Mental and Physical Health and Clinical Translation), Geelong, Victoria, Australia; Barwon Health, Geelong, Victoria, Australia; Department of Medicine - Western Health, The University of Melbourne, St Albans, Victoria, Australia; Department of Orthopedics and Traumatology, Kuopio University Hospital, Kuopio, Finland; Kuopio Musculoskeletal Research Unit, University of Eastern Finland, Kuopio, Finland; Department of Medicine and Therapeutics, Faculty of Medicine, The Chinese University of Hong Kong, Hong Kong; Jockey Club Centre for Osteoporosis Care and Control, Faculty of Medicine, The Chinese University of Hong Kong, Hong Kong; Centre of Bone Diseases, Lausanne University Hospital, Lausanne, Switzerland; Service of Internal Medicine, Lausanne University Hospital, Lausanne, Switzerland; HUNT Research Centre, Department of Public Health and Nursing, Faculty of Medicine and Health Sciences, Norwegian; University of Science and Technology, Trondheim, Norway; Endocrinology and Metabolism Research Center, Endocrinology and Metabolism Clinical Sciences Institute, Tehran University of Medical Sciences, Tehran, Iran; Department of Osteoporosis, Bern University Hospital, University of Bern, Bern, Switzerland; Geriatric Medicine, Department of Internal Medicine and Clinical Nutrition, Institute of Medicine, Sahlgrenska Academy, University of Gothenburg, Gothenburg, Sweden; Geriatric Medicine, Sahlgrenska University Hospital Mölndal, Mölndal, Sweden; Department of General Practice, Amsterdam UMC, location AMC, Amsterdam Public Health Research Institute, Amsterdam, The Netherlands; School of Sport Sciences, UiT The Arctic University of Norway, Tromsø, Norway; Department of Health Sciences, Swedish Winter Sports Research Centre, Mid Sweden University, Östersund, Sweden; Department of Medical Sciences, Uppsala University, Sweden; Department of public health and caring sciences, Uppsala University, Uppsala, Sweden; National Institute for Health Research Manchester Biomedical Research Centre, Manchester University NHS Foundation Trust, Manchester Academic Health Science Centre, Manchester, UK; Centre for Epidemiology Versus Arthritis, University of Manchester, Manchester, UK; Department of Internal Medicine, Division of Endocrinology and Diabetology, Medical University Graz, Graz, Austria; Center for Biomarker Research in Medicine, Graz, Austria; Sahlgrenska Osteoporosis Centre, Department of Internal Medicine and Clinical Nutrition, Institute of Medicine, Sahlgrenska Academy, University of Gothenburg, Gothenburg, Sweden; Department of Drug Treatment, Sahlgrenska University Hospital, Region Västra Götaland, Gothenburg, Sweden; Department of Medicine, Oregon Health and Science University, Portland, Oregon, USA; Deakin University, Institute for Physical and Mental Health and Clinical Translation (IMPACT), Geelong, Australia; Department of Medicine-Western Health, The University of Melbourne, St Albans, Australia; Barwon Health, Geelong, Australia; Department of Epidemiology and Preventive Medicine, Monash University, Melbourne, Australia; Department of Internal Medicine, Erasmus University Medical Center, Rotterdam, The Netherlands; Université Claude Bernard Lyon 1, U INSERM 1290 RESHAPE, Lyon, France; Laboratory of Epidemiology and Population Sciences, National Institute on Aging, Baltimore, Maryland, USA; Icelandic Heart Association, Kopavogur, Iceland; Janus Rehabilitation, Reykjavik, Iceland; Translational Gerontology Branch, National Institute on Aging Intramural Research Program, Baltimore, Maryland; INSERM UMR 1033, University of Lyon, Hôpital Edouard Herriot, Lyon, FranceINSERM; Kuopio Musculoskeletal Research Unit, University of Eastern Finland, Kuopio, Finland; Department of General Practice, Amsterdam UMC, location VUmc, Amsterdam Public Health Research Institute, Amsterdam, The Netherlands; PHARMO Institute for Drug Outcomes Research, Utrecht, The Netherlands; INSERM UMR 1033, University of Lyon, Hôpital Edouard Herriot, Lyon, France; Department of Hygiene and Public Health, Faculty of Medicine, Educational Foundation of Osaka Medical and Pharmaceutical University, Osaka, Japan; York Trials Unit, Department of Health Sciences, University of York, York, UK; Department of Epidemiology and Data Science, Amsterdam Public Health Research Institute, VU University Medical Center, Amsterdam, The Netherlands; Centre for Health Informatics, Faculty of Biology, Medicine and Health, School of Health Sciences, University of Manchester, Manchester, UK; Statistics Support Unit, Hospital del Mar Medical Research Institute, CIBER Epidemiology and Public Health (CIBERESP), Barcelona, Spain; MRC Epidemiology Unit, University of Cambridge, Cambridge, United Kingdom; Department of Epidemiology, University of Alabama at Birmingham, Birmingham, Alabama, USA; Department of Preventive Medicine for Locomotive Organ Disorders, The University of Tokyo Hospital, Tokyo, Japan; Department of Internal Medicine, Erasmus University Medical Center, Rotterdam, The Netherlands; Health Center Can Gibert del Plà, Catalan Institute of Health, Girona, Spain; Department of Medical Sciences, University of Girona, Girona, Spain; GROIMAP/GROICAP (research groups), Unitat de Suport a la Recerca Girona, Institut Universitari d’investigació en Atenció Primària Jordi Gol, Girona, Spain; PRECIOSA-Fundación para la investigación, Barberà del Vallés, Barcelona, Spain; Mary McKillop Institute for Health Research, Australian Catholic University, Melbourne, Australia; Sahlgrenska Osteoporosis Centre, Department of Internal Medicine and Clinical Nutrition, Institute of Medicine, Sahlgrenska Academy, University of Gothenburg, Gothenburg, Sweden; MRC Lifecourse Epidemiology Centre, University of Southampton, Southampton, UK; NIHR Southampton Biomedical Research Centre, University of Southampton and University Hospital Southampton NHS Foundation Trust, Southampton, UK; Mary McKillop Institute for Health Research, Australian Catholic University, Melbourne, Australia; Sahlgrenska Osteoporosis Centre, Institute of Medicine, University of Gothenburg, Sweden; Region Västra Götaland, Geriatric Medicine, Sahlgrenska University Hospital, Mölndal, Sweden; Department of Medicine, University of Manitoba, Winnipeg, Manitoba, Canada

**Keywords:** Prior fracture, Meta-analysis, Hip fracture, Osteoporotic fracture, Major osteoporotic fracture

## Abstract

**Summary:**

A large international meta-analysis using primary data from 64 cohorts has quantified the increased risk of fracture associated with a previous history of fracture for future use in FRAX.

**Introduction:**

The aim of this study was to quantify the fracture risk associated with a prior fracture on an international basis and to explore the relationship of this risk with age, sex, time since baseline and bone mineral density (BMD).

**Methods:**

We studied 665,971 men and 1,438,535 women from 64 cohorts in 32 countries followed for a total of 19.5 million person-years. The effect of a prior history of fracture on the risk of any clinical fracture, any osteoporotic fracture, major osteoporotic fracture and hip fracture alone was examined using an extended Poisson model in each cohort. Covariates examined were age, sex, BMD and duration of follow up. The results of the different studies were merged by using the weighted β-coefficients.

**Results:**

A previous fracture history, compared with individuals without a prior fracture, was associated with a significantly increased risk of any clinical fracture (Hazard ratio, HR = 1.88; 95% CI = 1.72-2.07). The risk ratio was similar for the outcome of osteoporotic fracture (HR = 1.87; 95% CI = 1.69-2.07), major osteoporotic fracture (HR = 1.83; 95% CI = 1.63-2.06) or for hip fracture (HR = 1.82; 95% CI = 1.62-2.06). There was no significant difference in risk ratio between men and women. Subsequent fracture risk was marginally downward adjusted when account was taken of BMD. Low BMD explained a minority of the risk for any clinical fracture (14%), osteoporotic fracture (17%), and for hip fracture (33%). The risk ratio for all fracture outcomes related to prior fracture decreased significantly with adjustment for age and time since baseline examination.

**Conclusion:**

A previous history of fracture confers an increased risk of fracture of substantial importance beyond that explained by BMD. The effect is similar in men and women. Its quantitation on an international basis permits the more accurate use of this risk factor in case finding strategies.

## Introduction

A history of a prior fracture at a site characteristic for osteoporosis is an important risk factor for further fracture [1, 2, 3, 4, 5, 6]. Fracture risk is approximately doubled in the presence of a prior fracture, including morphometric vertebral fractures. The risks are in part independent of BMD [4]. However, the increase in risk is not constant with age. For example, a large meta-analysis showed that a prior fracture history was a significant risk factor for hip fracture at all ages, but the population relative risk was highest at younger ages and decreased progressively with age [4].

The identification of patients with a fracture history is a well-established goal in the clinical management of osteoporosis as outlined in most clinical guidelines worldwide [7, 8, 9, 10, 11, 12]. In many cases, individuals with a prior fracture are eligible for treatment irrespective of BMD. For example, the National Osteoporosis Guideline Group (NOGG) in the United Kingdom recommends treatment in all women with a prior fragility fracture [10]. A similar threshold is provided in the European guidance [13]. In the United States, a prior vertebral or hip fracture qualifies for a treatment recommendation irrespective of BMD [14].

Because a prior fracture provides a fracture risk that is largely independent of BMD, it has been incorporated into assessment guidelines that integrate the risks associated with a number of risk variables [15, 16, 17]. FRAX®, currently available in 78 territories, is the most widely used fracture risk assessment tool and is incorporated into a large number of assessment guidelines [7], recommended by the Committee for Medicinal Products for Human Use (CHMP) [18], and approved by the National Institute for Health and Care Excellence (NICE) [19]. The incorporation of a prior fracture as an input variable for risk prediction was based on a meta-analysis, published in 2004, of 15,259 men and 44,902 women from 11 cohorts followed for a total of 250,000 person-years [4]. Since then, many more prospectively studied cohorts have become available that have the potential to improve the accuracy of FRAX [20].

The aim of the present study was to quantify the risk for future fracture associated with a history of prior fracture in an international setting, and to explore the dependence of this risk on age, sex, time since baseline assessment and BMD.

## Methods

The study population was derived from a systematic review that identified prospective cohort studies for the update of FRAX. The study was registered with the International prospective register of systematic reviews, PROSPERO (CRD42021227266), and followed the Preferred Reporting Items for Systematic Reviews (PRISMA) guidelines. Studies were eligible if the cohort was prospective, included at least 200 participants, assessed an adequate number of clinical risk factors and reported an adequate number of incident fracture outcomes. We studied 2,104,506 men and women from 64 prospectively studied cohorts of whom 9.7% had a prior fracture history. 58 cohorts included women (n=1,438,535) and 40 cohorts included men (n=665,971). Details of the cohorts studied have been given previously [20] and are summarized in Table 1.

### Baseline and outcome variables

The construct of the question to determine a prior fracture history differed between the cohorts studied, based on time of previous fracture, fracture site, energy, validity, and inclusion of morphometric vertebral fractures (Table 2).

For outcomes Information on all clinical fractures was used for this report ‘all fractures’. In addition, fractures considered to be associated with osteoporosis were examined [21]. According to this classification, fractures of the skull, face, hands, feet, ankle and patella were excluded as well as tibial and fibular fractures in men. Hip fracture and major osteoporotic fracture were also analysed separately. No distinction was made according to trauma since both high- and low-trauma fractures show similar relationships with low BMD and future fracture risk [22]. The risk of death as function of fracture history was also assessed.

### Statistical methods

The risk of fracture was estimated by an extended Poisson model applied separately to each cohort (and also separately by sex for those cohorts with both men and women) [23, 24]. Because of an embargo on transfer of primary data from Manitoba, Cox regression was used on the Manitoba cohort on site and beta-coefficients, variances and covariances forwarded to the analysis team. Covariates included current time since start of follow up, current age (derived from age at since start of follow up and current time since start of follow up), prior history of fracture, and BMD at the femoral neck. Femoral neck BMD was adjusted for manufacturer and T-scores were calculated from the NHANES III White female reference values [20]. We additionally estimated a model that excluded BMD from the covariates. A further model included the interaction term ‘prior fracture · current time since baseline’ to determine whether the strength of the association of prior fracture and fracture risk changed with time. An additional model included the interaction term ‘prior fracture · current age’ to determine whether the strength of the association of prior fracture and fracture risk changed with age. Interactions with time and with age were also explored using piece-wise linear regression to check the adequacy of the Poisson model. The hazard ratio (HR) for previous fracture was determined for each age from 40 years from the Poisson model. Results of each cohort and the two sexes were weighted according to the variance and merged to determine the weighted means and standard deviations. The HR of those with a prior fracture history versus those without a prior fracture history was equal to e^weighted mean of β^. There was significant heterogeneity in risk between cohorts (index of heterogeneity I^2^ = 82-98% depending on fracture outcome), and a random effects model was used in the meta-analysis.

The component of the risk ratio explained by BMD was computed from a meta-analysis of BMD and fracture risk in men and women combined [25]. Based on the prior evidence, the risk of any clinical fracture was assumed to increase 1.45-fold for each SD decrease in BMD at the femoral neck. For hip fracture, the gradient of risk was assumed to be 2.07 per SD and 1.55 for any osteoporotic fracture [4]. These findings permitted comparison of the calculated expected difference in mean BMD between those with, versus those without, a prior fracture, with the actual difference ascertained from the baseline data. Thus, the proportion of risk attributed to a low BMD was computed as:

$$\frac{\left[\log{\text{HR}}_{\text{a}}/\log\text{GR}\right]-\left[\log{\text{HR}}_{\text{b}}/\log\text{GR}\right]}{\left[\log{\text{HR}}_{\text{a}}/\log\text{GR}\right]}$$

where HR_a_ is the unadjusted hazard ratio for prior fracture, HR**b** is the hazard ratio adjusted for BMD, and GR is the gradient of risk for femoral neck BMD [4].

Individuals with missing data were excluded. No data were imputed.

### Sensitivity analyses

As noted above, the effect of sex on the hazard ratio for fracture was examined in those cohorts that contributed both men and women. Similarly, differences in risk with and without BMD were additionally explored in those cohorts that contributed both scenarios. Assessment of the effects of race and ethnicity was confined to those cohorts recording more than one race or ethnic group (Asian, Black, Hispanic, White), comprising Health ABC, CAMOS, MROs USA, WHI, SOF, Manitoba and UK Biobank. Results were also computed according to study quality as previously defined [20]. Quality was based on a 0/1 score for four criteria: Population-based cohort (yes scores 1); Fracture ascertainment (self-report scores 0, others score 1); Duration of follow-up (> 2 years, scores 1); Average loss to follow-up/year (< 10%, scores 1). This gives a maximum score of 4 and a minimum of 0. A quality score of 0 or 1 was designated as poor quality (designated C), a score of 2 or 3 categorised as intermediate quality (B) and a score of 4 designated as high quality (A). Quality grades are given in Table1.

## Results

Of 2,104,506 men and women studied in 32 countries, 45,059 men and 158,659 women had sustained a prior fracture. At follow up, 38,897 men and 147,897 women were identified as having a subsequent clinical fracture of any kind; 31,686 and 124,139 were characterized as osteoporotic in men and women, respectively; 26,744 men and 83,815 women sustained a MOF; 8182 and 31,176 were hip fractures. The total follow-up time was 6.8 million-person years in men and 12.7-million-person years in women. BMD measurements were available in 13.8% (289,841) of individuals. The probability of fracture history rose almost linearly with age from the age of 40 years but tended to decline in women after age 90 years (Table 3). The prevalence of recording a history of a prior fracture was higher in women than in men (OR = 1.34; 95% CI = 1.32–1.35 unadjusted).

### Risk of fracture by site and sex

Previous fracture was associated with a significantly increased risk of any subsequent fracture (Table 4). In men and women, the HR ranged from 1.71 to 1.99 depending upon category of the outcome fracture. There were no significant differences in hazard ratios by site of fracture. The risk ratio was marginally but not significantly higher in men than in women by approximately 7-11%. In a sensitivity analysis using only those cohorts that contributed both men and women, there was no sex difference in hazard ratio for all sites (Appendix, Table A)

**Figure 1:**
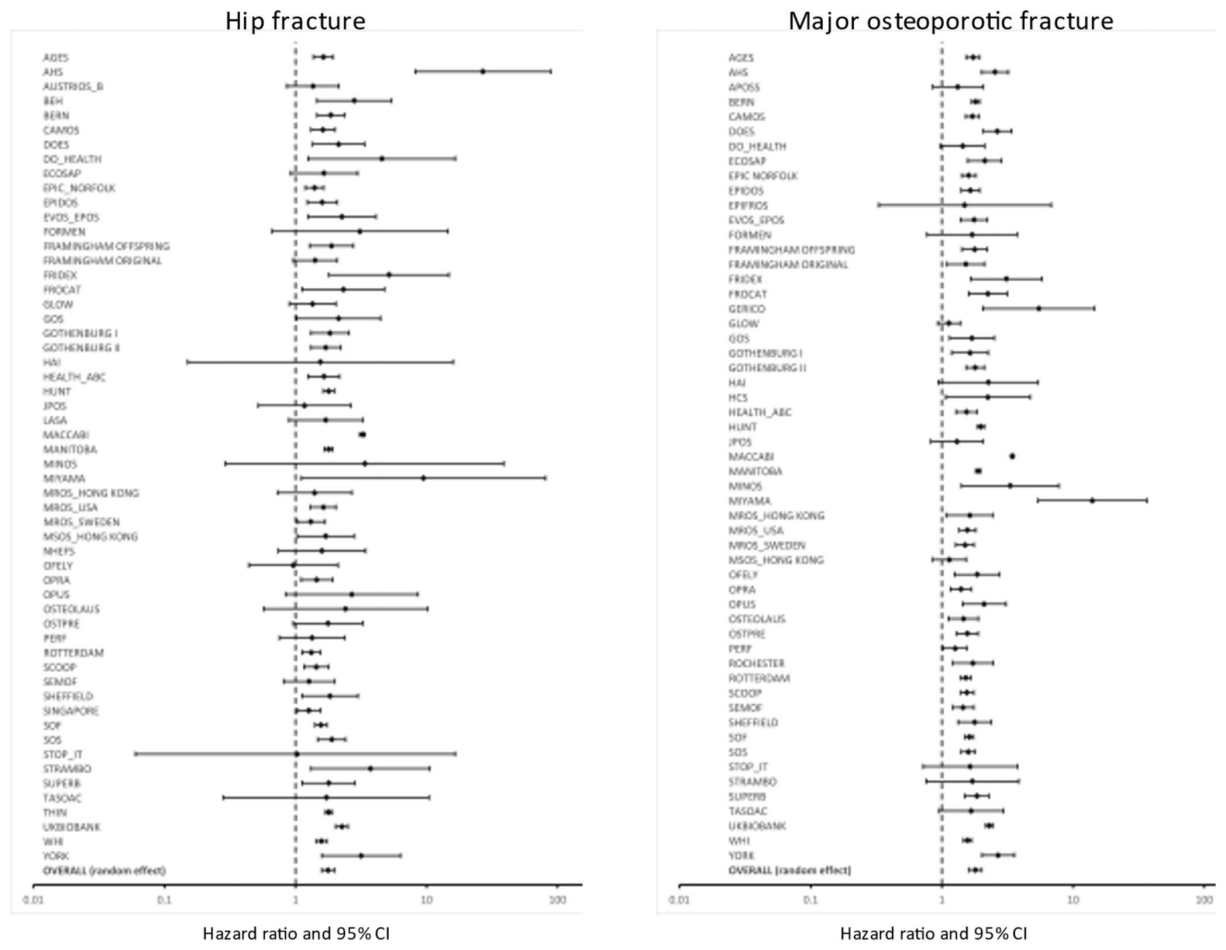
Forest plot showing effect size on hip fracture risk (left panel) and major osteoporotic fracture (right panel) associated with a prior fracture in men and women combined adjusted for age and time since baseline

The increase in risk among those who reported a prior clinical fracture was fairly heterogeneous as shown in the Forest plots in Figure 1 for MOF and hip fracture outcomes. Forest plots for any clinical fracture and osteoporotic fracture outcomes are given in the appendix. Heterogeneity was not related to the question construct since the question construct had little effect on the outcome. In the case of an osteoporotic fracture, for example, the question construct of any prior fracture was associated with a similar increase in fracture risk (HR=1.87; 95%CI=1.58-2.22) as that when the question referred to a prior major osteoporotic fracture (HR=1.77; 95%CI=1.51-2.07) or where the site of prior fracture was unspecified (HR=1.75; 95%CI=1.61-1.89). Similarly, there was no significant difference when low or moderate trauma was specified (HR=1.77; 95%CI=1.41-2.22) or unspecified (HR=1.84; 95%CI=1.67-2.03; p>0.3).

### Dependence on BMD

The impact of BMD on the fracture risk in individuals with a prior fracture is quantified in Table 5. The HR was marginally decreased by approximately 8-16% when account was taken of BMD. In the case of any clinical fracture, if it is assumed that the risk of any clinicalfracture increases 1.45-fold for each standard deviation (SD) decrease in hip BMD (gradient of risk), then the difference in risk between those with and without a prior fracture is equal to an expected difference in BMD of 1.57SD [log 1.79/log1.45]. In reality, the difference in BMD at all ages in men and women combined was approximately 0.22 SD ([log (1.79)/log(1.45)]- [log(1.65)/log(1.45)]). Thus, low BMD accounted for the minority (14%; 0.22/1.57) of the difference in risk of any clinical fracture between those with or without a prior fracture. As would be expected, the proportion of risk accounted for by BMD was greater in the case of hip fractures (see Table 5) but remained less than 50% (see Table 5).

### Interaction with age

**Figure 2:**
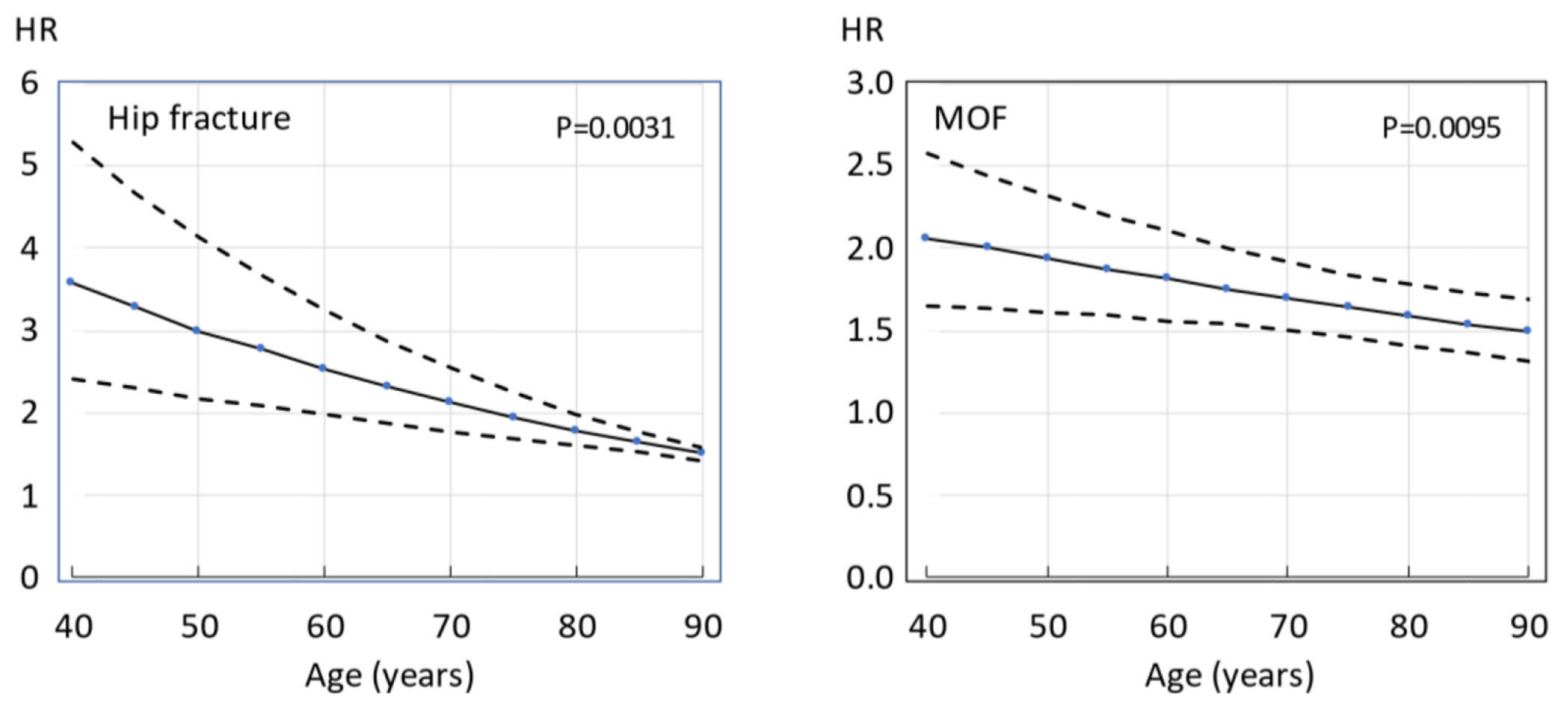
Hazard ratio (HR) and 95% confidence interval of a major osteoporotic fracture (MOF) and hip fracture by age associated with a history of prior fracture in men and women combined. HRs are adjusted for time since baseline and sex.

A prior fracture history was a significant risk factor for fracture at all ages. The hazard ratio was highest at younger ages and decreased progressively with age (Table 6). The interaction term was significant for all fracture outcomes in men and women combined. The decrease with age was most marked for hip fracture which decreased by approximately 16% for each decade of age (Figure 2). An almost identical relationship was observed using piece-wise linear regression (data not shown).

### Interaction with time

Fracture risk associated with a prior fracture decreased slowly with time since baseline by about 2-4% per year (Table 7). A similar relationship was observed using piece-wise linear regression (data not shown).

### Race and Ethnicity

With one exception, there was no difference in the HR by race and ethnicity in those cohorts where race or ethnicity was documented (Table B of Appendix). The exception was for major osteoporotic fracture such that in Blacks, those with prior fracture history had a higher risk of subsequent fracture hazard ratio than Whites (Blacks: HR=2.43, 95% CI=1.37-3.78 vs. Whites: HR=1.57, 95% CI= 1.32-1.87). The effect was largely driven by a high HR in Blacks from Manitoba (HR=5.34, 95% CI= 1.79-15.94).

### Quality scores

There was no significant difference in fracture outcomes when cohorts of high quality were compared with those of moderate quality (Appendix, Table C). For cohorts of low quality, there was a significant difference from high quality cohorts for MOF, based on a single low-quality cohort (GERICO).

### Risk of death

A prior fracture was associated with a significant increase in the risk of death in both men (HR=1.11; 95%CI=1.02, 1.21) and women (HR=1.10; 95%CI=1.05-1.15). Hazard ratios remained unchanged when adjusted for femoral neck BMD.

## Discussion

The present study represents the largest meta-analysis to date on the association between prior fracture and subsequent fracture risk. The effect is similar in men and women and is consistent with our previous meta-analyses [4]. It is of interest that the quantum of effect was not dependent the question construct. The size of the effect was also relatively immune to cohort quality and different race and ethnicities. Nonetheless, the true effect size relies on the accuracy of information provided which cannot be assessed in the construct of the present study. For the purposes of risk assessment, however, accuracy and causality of associations are of less concern than repeatability and that the risk identified shows reversibility of effect [17, 28].

The extensive data resource permitted the elucidation of important interactions comprising an interaction with age, and time since baseline. For all fracture outcomes, the risk ratios decreased significantly with age, consistent with our previous meta-analysis [4] and incorporated into FRAX [17]. Of Importance, we were able to examine the risk associated with prior fractures among the oldest -old. Additionally, the increased power of the present study revealed that hazard ratios also decreased significantly with time, a phenomenon not accounted for in the current FRAX model [17]. As with all risk variables used in FRAX, any interaction of effect over time is also important to incorporate in future probability models.

The present study also quantified the independent contributions of low BMD and prior fracture. For all outcomes studied, low BMD explained a minority of the total risk. The mechanism for the BMD-independent increase in risk could not be determined from this study but is likely due, in part, to coexisting morbidity that might increase the risk of falls or impair the protective responses to injury [27, 28]. In addition, changes in the structural or material properties of bone may weaken bone out of proportion to any effect on BMD [29, 30, 31, 32, 33, 34].

A particular strength of the present study is that the estimate of risk is made in an international setting largely from population-based cohorts. Calculations were based on the primary data, decreasing the risk of publication biases. The consistency of the association between cohorts additionally indicates the international validity of this risk variable. The present study has several limitations that should be mentioned. As with nearly all population-based studies, nonresponse biases may have occurred, which we were unable to document for all cohorts. The effect is likely to exclude sicker members of society, including those in institutional care, and may underestimate the absolute risk of fracture. Thus, the probability of a prior fracture may be underestimated from a societal perspective, but this is unlikely to affect risk ratios. The greatest potential problem was the construct of the question concerning prior fractures and the methods of documenting and characterizing subsequent fracture events. These differed substantially between cohorts. The effect of this heterogeneity on fracture outcomes was, however, marginal. It should also be recognised that additional factors affect the risk associated with a prior fracture. The increase in risk is more marked the greater the number of prior fractures [35, 36, 37], particularly prior vertebral fractures for a subsequent vertebral fracture [35, 38, 39, 40, 41]. Also, the risk of a subsequent osteoporotic fracture is particularly acute immediately after an index fracture and wanes progressively with time [3, 42, 43, 44]. For example, after a fracture, the risk of subsequent fracture is highest in the immediate post fracture interval with more than one-third of subsequent fractures occurring within 1 year [45]. The waning of risk with time is also age-dependent [44]. Also, the effect of recency is site dependent [47] with higher risk ratios for hip and vertebral fracture than for humerus, forearm, or minor osteoporotic fracture. Finally, morphometric but subclinical fractures were not assessed though they do add to fracture probability independently of FRAX [48]. Data on these additional modulating factors were not available for this meta-analysis, thus residual confounding could be present in our findings. However, adjustments to FRAX probabilities for these factors is available through FRAXplus [49]. FRAXplus, which has recently been released in a beta version, brings together a number of adjustments that can illustrate the potential impact of modulating factors on FRAX fracture probabilities. These include trabecular bone score, recency of fracture (by site and time within the last two years), the number of self-reported falls in the previous year, glucocorticoid dose, and duration of type 2 diabetes mellitus. An additional limitation is that no account was taken of treatment effects.

In conclusion, this analysis has quantified the magnitude of the risk for future fractures conferred by a prior fracture in the largest meta-analysis conducted to date, and that this risk is largely independent of BMD. The effect is similar in men and women. The consistency of the association in an international setting provides the rationale for the use of these data in the next iteration of FRAX.

## Supplementary Material

Supplementary Material

## Figures and Tables

**Figure 3:**
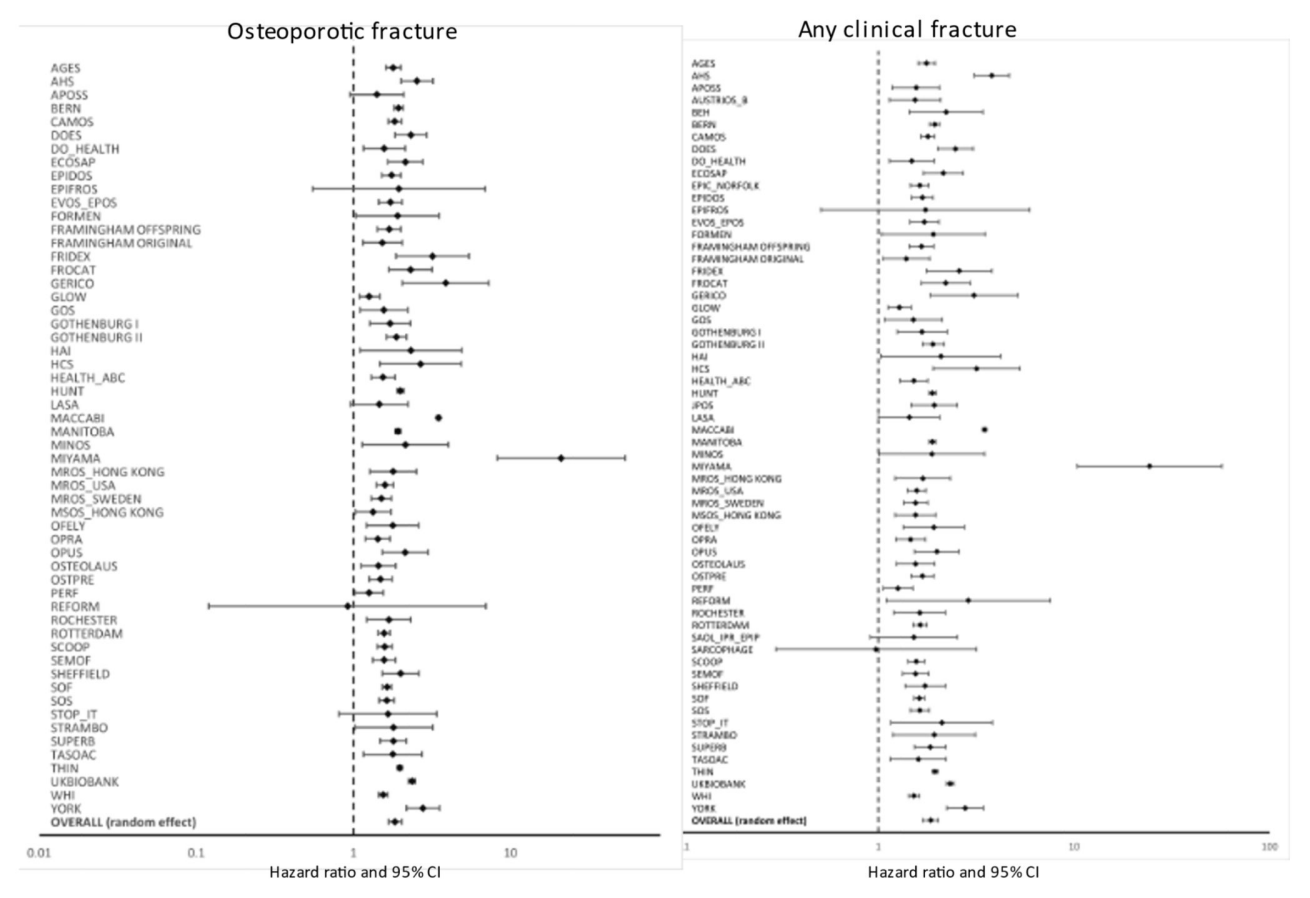
Forest plot showing effect size on osteoporotic fracture risk (left panel) and any clinical fracture (right panel) associated with a prior fracture in men and women combined adjusted for age and time since baseline

**Table 1:** Characteristics of the cohorts studied

	Quality grade			Age (years)				Number of fractures				
Cohort		n	Person years	Mean	Range	% female	Prior fracture (%)	Hip	Any	MOF	MOF minus hip	Osteoporotic
AGES	A	5706	45508	77.0	66 -98	57.6	42.2	535	1619	1134	766	1395
AHS	B	2613	10109	65.1	47-95	69.6	25.9	32	368	281	257	281
APOSS	A	3840	33629	48.5	44-56	100	13.1	4	335	142	141	176
AUSTRIOS B	C	2046	2370	83.9	68-103	84.1	46.6	76	174	-	-	-
BEH	B	2414	10085	69.3	60-96	51.9	12.9	42	105	-	-	-
Bern	B	23104	181352	58.9	20-95	85.0	43.9	294	5033	2913	2730	3891
CaMos	A	9422	121627	62.1	25-103	69.4	44.0	340	2435	1188	935	1753
DO_HEALTH	B	2139	5914	75.0	70-95	61.9	22.5	10	264	118	111	190
DOES	A	2133	18884	70.1	47-94	60.7	15.0	110	561	363	294	465
ECOSAP	B	5146	16857	72.3	65-100	100	20.2	52	311	188	136	259
EPIC-Norfolk	A	25600	493500	59.2	39-79	54.7	7.0	1356	3040	2344	1205	-
EPIDOS	B	7595	21192	80.5	70-100	100	45.0	226	1026	568	376	837
EPIFROS	B	284	2826	61.6	40-96	54.6	4.6	3	27	16	13	20
EVOS/EPOS	B	13366	40983	63.8	41-91	52.1	36.3	44	538	286	245	538
FORMEN	A	1885	16253	72.5	65-93	0	7.9	10	90	58	49	90
Framingham offspring	A	3539	58402	61.5	33-90	54.1	33.9	105	758	316	239	533
Framingham original	A	1166	11184	79.9	72-101	65.3	20.0	136	279	187	68	242
FRIDEX	B	815	8077	56.8	40-84	100	20.4	15	112	41	28	56
FROCAT	A	1953	19404	69.2	32-111	55.7	17.1	33	229	160	135	183
GERICO	C	764	2766	67.9	65-72	79.5	46.3	2	71	26	24	51
GLOW	B	54258	216703	68.2	55-108	100	3.1	490	5690	2848	2437	4285
GOS	A	1403	9364	69.5	50-95	100	30.3	31	149	105	80	135
Gothenburg I	A	1736	9818	85.5	70-96	57.0	10.7	304	431	361	100	408
Gothenburg II	A	11371	149825	59.0	21-84	100	16.8	259	1192	739	644	856
HAI	B	2085	3303	70.5	70-72	51.1	14.1	4	42	26	22	36
HCS	A	632	5595	64.9	59-71	50.3	16.3	3	67	35	33	51
Health ABC	A	3062	36309	73.6	68-80	51.5	22.0	235	696	518	349	594
HUNT	A	50209	622020	53.2	20-100	54.6	23.4	1674	10239	4733	3601	7128
JPOS	B	1944	25812	57.5	40-82	100	15.8	29	265	99	-	-
LASA	A	1473	7575	75.7	65-89	51.6	27.9	38	131	-	-	95
Maccabi	A	659266	6297325	56.3	30-91	52.0	4.8	11293	54312	51955	42759	53907
Manitoba	B	92281	833424	63.4	20-104	89.1	21.3	3085	13506	9578	7187	12655
MINOS	B	681	6152	65.2	50-86	0	12.8	3	63	25	22	56
Miyama	A	400	3703	59.1	40- 79	50.0	33.5	7	61	35	30	47
MrOS Hong Kong	B	2000	19744	72.4	65-92	0	13.7	63	231	148	93	201
MrOS Sweden	A	2999	34019	74.9	69-81	0	20.9	339	968	728	482	874
MrOS USA	A	5993	74998	73.7	64-100	0	55.3	330	1394	814	490	1082
MsOS Hong Kong	B	2000	17528	72.6	65-98	100	20.8	69	338	247	189	298
NHEFS	A	12206	121623	49.4	25-74	59.6	6.7	113	-	-	-	-
OFELY	A	867	15136	58.8	40-89	100	10.3	40	245	180	159	207
OPRA	A	1044	12133	75.2	75-76	100	45.8	195	524	453	-	473
OPUS	B	1983	12167	62.0	20-80	100	42.0	14	236	113	102	148
OsteoLaus	B	1475	6726	64.5	50-82	100	36.4	8	307	226	221	245
OSTPRE	B	11200	109465	57.3	52-62	100	9.0	80	1851	918	848	1259
PERF	B	5760	37802	64.2	44-81	100	17.3	62	828	544	489	550
REFORM	C	1003	1483	77.9	65- 99	60.5	6.5	4	30	12	8	17
Rochester	A	1001	7686	56.8	21-94	65.2	18.1	37	326	243	229	283
Rotterdam	A	14619	158085	65.8	45-106	58.8	22.9	830	3317	2322	1742	2892
SAOL_IPR_EPIPorto	B	929	11284	55.9	40- 89	77.4	12.7	12	105	9	-	-
SarcoPhAge	C	228	440	75.9	68-93	57.0	25.4	1	13	5	4	8
SCHS	A	52042	462436	61.6	48- 84	57.4	8.1	1091	-	-	-	-
SCOOP	A	12368	58826	75.6	70-86	100	23.1	378	1927	1284	975	1625
SEMOF	B	7130	20624	75.2	70 -91	100	51.7	80	683	464	384	596
Sheffield	B	2148	7354	80.0	74-101	100	45.4	66	281	186	132	227
SOF	B	9619	135474	71.6	65-89	100	37.1	1404	4337	2794	1833	3455
SOS	B	16626	62119	74.2	61-92	100	30.0	260	1383	993	702	1325
STOP/IT	B	424	1840	71.1	65-87	55.0	49.1	2	50	24	22	32
STRAMBO	A	823	7582	72.1	51-88	0	11.7	17	117	42	26	86
SUPERB	B	3019	10736	77.8	75-81	100	36.8	70	463	341	-	421
TASOAC	B	1098	10955	63.0	51-81	48.9	44.2	5	146	49	46	88
THIN	A	366104	2125764	63.8	50-116	100	9.1	6942	31633	-	-	23622
UK Biobank	B	502536	5766212	56.5	37-73	54.4	3.7	3943	25190	12099	8332	20075
WHI	B	64399	868380	65.8	55-79	100	17.4	1981	5259	3712	1901	4213
York	B	4532	9044	77.1	48-99	100	44.7	42	393	223	189	310
Total		2104506	19535515		20-116			39358	186794	110559	84614	155825?
Mean				61.5		68.3	9.7					

MOF, major osteoporotic fracture; AGES, Age, Gene/Environment Susceptibility-Reykjavik Study; AHS, Adult Health Study; APOSS, Aberdeen Prospective Osteoporosis Screening Study; BEH, Bushehr Elderly Health; CaMos, Canadian Multicentre Osteoporosis Study; DOES, Dubbo Osteoporosis Epidemiology Study; DO-HEALTH, VitaminD3-Omega3-Home Exercise-Healthy Aging and Longevity Trial; ECOSAP, Ecografía Osea en Atención Primaria; EPIC-Norfolk, European Prospective Investigation of Cancer-Norfolk; EPIDOS, Epidémiologie de l’Ostéoporose; EPIFROS, EPIdemiology and Fracture Risk factors for Osteoporosis in Spain; EVOS/EPOS, European Vertebral Osteoporosis Study/European Prospective Osteoporosis Study; FORMEN, Fujiwara-kyo Osteoporosis Risk in Men; FRIDEX, Fracture RIsk factors and bone DEnsitometry type central dual X-ray; FROCAT, Fracture Risk factors for Osteoporosis in CATalonia; GERICO, Geneva Retirees Cohort; GLOW, Global Longitudinal Study of Osteoporosis in Women; GOS, Geelong Osteoporosis Study; HAI, Healthy Ageing Initiative; HCS, Hertfordshire Cohort Study; Health ABC, Health, Aging and Body Composition; HUNT, The Trøndelag Health Study; JPOS, Japanese Population-based Osteoporosis Study; LASA, Longitudinal Aging Study Amsterdam; MINOS, Montceau les MINes OSteoporosis; MrOS, Osteoporotic Fractures in Men; MsOS, Osteoporotic Fractures in Women; NHEFS, National Health and Nutrition Examination Survey (NHANES) I Epidemiologic Follow-up Study; OFELY, Os des Femmes de Lyon; OPRA, Osteoporosis Prospective Risk Assessment; OPUS, Osteoporosis and Ultrasound Study; OSTPRE, Kuopio OSTeoporosis risk factor and PREvention study; PERF, Prospective Epidemiologic Risk Factor; REFORM, REducing Falls with ORthoses and a Multifaceted podiatry intervention; SAOL-IPR-EPIPorto, Santo António dos Olivais, Instituto Português de Reumatologia and EPIPorto; SarcoPhAge, Sarcopenia and Physical Impairment with advancing Age; SCHS, Singapore Chinese Health Study; SCOOP, screening for prevention of fractures in older women; SEMOF, Swiss Evaluation of the Methods of Measurement of Osteoporotic Fracture risk; SOF, Study of Osteoporotic Fractures; SOS, SALT Osteoporosis Study; STRAMBO, Structure of the Aging Men’s Bone; SUPERB, Sahlgrenska University hospital Prospective Evaluation of Risk of Bone fractures; TASOAC, Tasmanian Older Adult Cohort; THIN, The Health Improvement Network; WHI, Women’s Health Initiative.

**Table 2:** Details of the construct of the questionnaire on fracture type and history in the cohorts studied.

Element	Construct
Time horizon	Ever in life, adult life, from age 18, 20, 35, 40, 45, 50, past 12 months, 5 years or10 years
Site of fracture	Any fracture, osteoporotic fracture, MOF
Energy	All trauma included, moderate trauma, low trauma
Validity	Self-reported, verified, based on GP medical record, administrative healthcare data, has a doctor/nurse/physician assistant told you?
Vertebral deformity	Vertebral fractures assessed by semiquantitative criteria included, not included

**Table 3:** Prevalence of a prior fracture history in men and women by age. The Manitoba and Maccabi data are not included since primary data were not available.

Age (years)	Fracture history (%)		
	Men	Women	Combined
40-49	4.2	3.5	3.8
50-59	5.9	7.0	6.6
60-69	6.4	11.0	9.6
70-79	14.1	20.6	19.3
80-89	17.8	23.7	22.7
90+	21.4	21.8	21.8

**Table 4:** Hazard ratio (HR) and 95% confidence interval (CI) of fracture at the sites indicated associated with a history of prior fracture in men and women and both sexes combined. HRs are adjusted for age and time since baseline.

	Outcome fracture	Number of cohorts	I^2^(%)	HR	95% CI
Women					
	Any	56	94	1.84	1.72-1.97
	Hip	51	81	1.71	1.57-1.86
	MOF	50	94	1.77	1.63-1.93
	MOF without hip fracture	45	91	1.80	1.65-1.95
	Osteoporotic	51	94	1.82	1.70-1.96
Men					
	Any	34	97	1.92	1.56-2.34
	Hip	29	91	1.99	1.53-2.59
	MOF	31	96	1.90	1.51-2.39
	MOF without hip fracture	30	94	1.79	1.43-2.25
	Osteoporotic	31	97	1.92	1.55-2.38
Men and women					
	Any	62	98	1.85	1.69-2.02
	Hip	56	92	1.77	1.59-1.98
	MOF	55	97	1.80	1.61-2.01
	MOF without hip fracture	51	96	1.80	1.62-2.01
	Osteoporotic	56	98	1.84	1.68-2.03

**Table 5:** Hazard ratio (HR) and 95% confidence interval (CI) of fracture at the sites indicated associated with a history of prior fracture in men and women combined. HRs are adjusted for age and time since baseline and additionally adjusted for BMD where indicated. The last column indicates the proportion of risk explained by BMD.

		Unadjusted		Adjusted for BMD			
Outcome fracture	Number of cohorts	HR	95% CI	HR	(95% CI)	Gradient of risk (HR/SD) for BMD	Proportion of risk (%) from BMD
Any	52	1.79	1.67-1.92	1.65	1.53-1.78	1.45	14
Hip	45	1.70	1.58-1.84	1.43	1.30-1.56	2.07	33
Osteoporotic	48	1.78	1.65-1.92	1.61	1.48-1.75	1.55	17

**Table 6:** Hazard ratio (HR) and 95% confidence interval (CI) of fracture by age at baseline at the sites indicated associated with a history of prior fracture in men and women combined. HRs are adjusted for time since baseline and sex. n refers to the number of cohorts available. P values refer to the significance of the interaction term with age

	Site of outcome fracture										
	Any (n=62)			Hip (n=56)			MOF (n=55)			Osteoporotic (n=56)	
Age (years)	HR	95% CI		HR	95% CI		HR	95% CI		HR	95% CI
40	2.47	1.96-3.13		3.57	2.42-5.27		2.32	1.77-3.03		2.40	1.87-3.08
45	2.38	1.93-2.94		3.27	2.30-4.67		2.22	1.74-2.84		2.31	1.84-2.89
50	2.29	1.90-2.76		3.00	2.18-4.13		2.13	1.71-2.66		2.22	1.82-2.72
55	2.20	1.87-2.59		2.76	2.08-3.66		2.05	1.68-2.49		2.14	1.79-2.55
60	2.11	1.84-2.43		2.53	1.98-3.24		1.97	1.66-2.33		2.06	1.76-2.40
65	2.03	1.81-2.28		2.32	1.88-2.86		1.89	1.63-2.19		1.98	1.73-2.25
70	1.96	1.78-2.15		2.13	1.78-2.54		1.81	1.60-2.05		1.90	1.71-2.12
75	1.88	1.75-2.02		1.95	1.70-2.25		1.74	1.57-1.92		1.83	1.68-1.99
80	1.81	1.72-1.90		1.79	1.61-1.99		1.67	1.55-1.80		1.76	1.65-1.88
85	1.74	1.68-1.80		1.64	1.52-1.77		1.60	1.52-1.69		1.69	1.62-1.77
90	1.67	1.63-1.72		1.51	1.43-1.59		1.54	1.49-1.59		1.63	1.58-1.68
		**P=0.0014**			**P<0.001**			**P=0.0011**			**P=0.0013**

**Table 7:** Hazard ratio (HR) and 95% confidence interval (CI) of fracture by time since baseline at the sites indicated associated with a history of prior fracture in men and women combined. HRs are adjusted for age and sex. N refers to the number of cohorts available. P values refer to the significance of the interaction term with time since baseline.

	Site of outcome fracture										
	Any (n=61)			Hip (n=54)			MOF (n=54)			Osteoporotic (n=55)	
Time (years)	HR	95% CI		HR	95% CI		HR	95% CI		HR	95% CI
0	2.12	1.78-2.52		2.12	1.73-2.69		2.06	1.65-2.57		2.13	1.76-2.58
1	2.06	1.76-2.41		2.04	1.70-2.55		2.00	1.63-2.44		2.07	1.74-2.45
2	2.00	1.73-2.30		1.97	1.68-2.42		1.93	1.61-2.32		2.00	1.71-2.33
3	1.94	1.71-2.20		1.91	1.65-2.30		1.87	1.59-2.20		1.94	1.69-2.23
4	1.88	1.68-2.11		1.84	1.63-2.19		1.81	1.56-2.10		1.88	1.66-2.13
5	1.83	1.65-2.02		1.78	1.59-2.08		1.75	1.54-2.00		1.82	1.62-2.03
6	1.77	1.61-1.95		1.72	1.56-1.99		1.70	1.50-1.92		1.76	1.58-1.95
7	1.72	1.58-1.88		1.66	1.52-1.91		1.64	1.46-1.84		1.70	1.54-1.89
8	1.67	1.53-1.83		1.60	1.48-1.84		1.59	1.41-1.78		1.65	1.49-1.83
9	1.62	1.48-1.78		1.55	1.42-1.78		1.54	1.37-1.73		1.60	1.43-1.78
10	1.58	1.43-1.74		1.49	1.37-1.73		1.49	1.31-1.69		1.55	1.38-1.74
		**P=0.0035**			**P=0.0031**			**P=0.0095**			**P=0.0042**
