# Supplementary material for: Previous fracture and subsequent fracture risk: A meta-analysis to update FRAX
Source: Osteoporos Int. Author manuscript; Available in PMC 2023 Dec 1. (PMC7615305; doi:10.1007/s00198-023-06870-z)
Supplement: Supplementary Material [file EMS187236-supplement-Supplementary_Material.pdf]

# Appendix

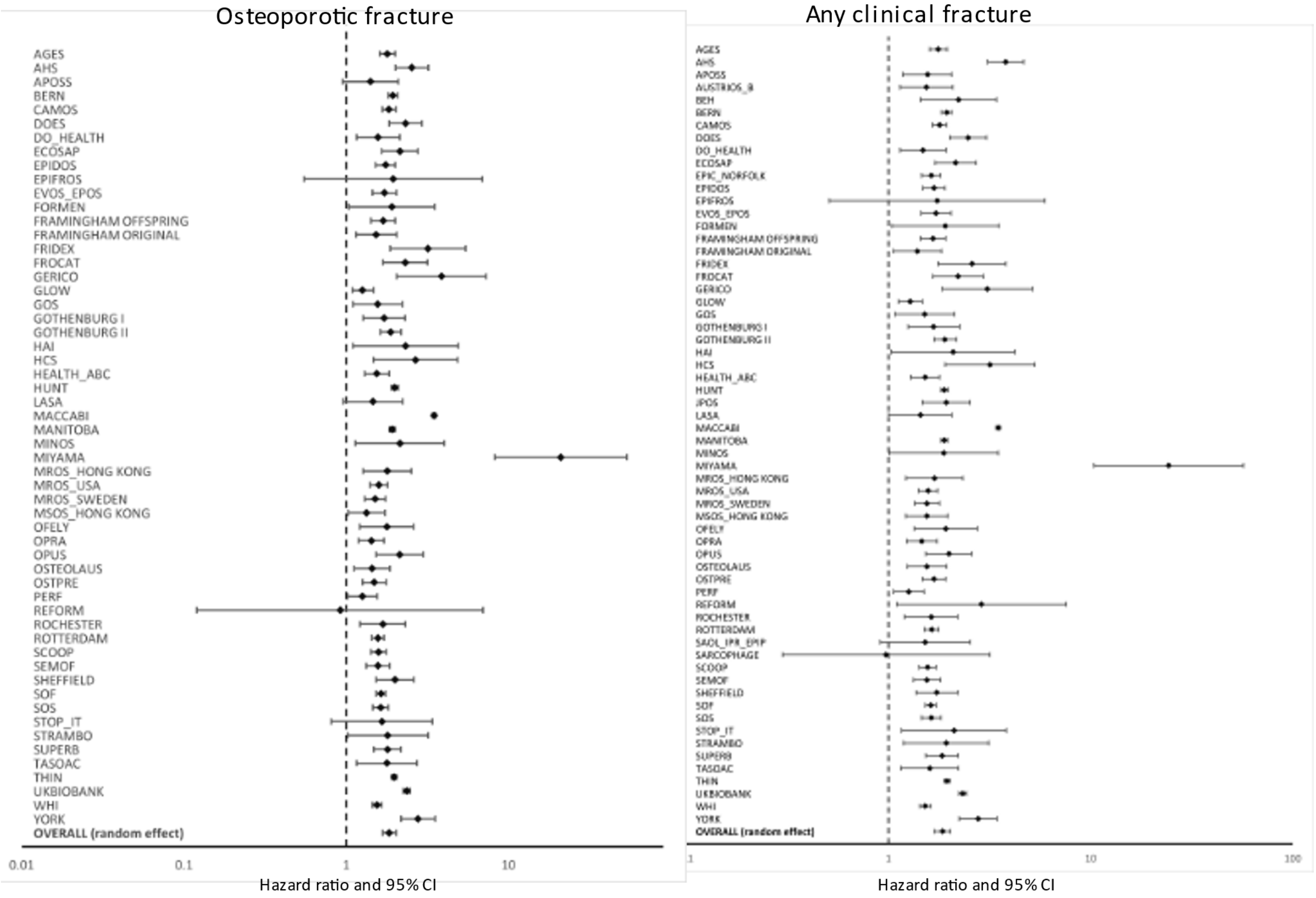


**Figure 3.** Forest plot showing effect size on osteoporotic fracture risk (left panel) and any clinical fracture (right panel) associated with a prior fracture in men and women combined adjusted for age and time since baseline.

**Table A**. Hazard ratio (HR) and 95% confidence interval (CI) at the sites shown associated with a history of a prior fracture in men and women in those cohorts that contributed both men and women

| Adjusted for BMD | Outcome fracture | Men | | | | Women | | | Number of cohorts | p-value for interaction |
|---|---|---|---|---|---|---|---|---|---|---|
| | | HR | 95% CI | | | HR | 95% CI | | | |
| No | Any | 1.94 | 1.55 | 2.42 | | 1.93 | 1.70 | 2.20 | 28 | 0.95 |
| | Hip | 1.94 | 1.44 | 2.61 | | 1.73 | 1.46 | 2.05 | 22 | 0.21 |
| | MOF | 1.90 | 1.48 | 2.45 | | 1.86 | 1.60 | 2.16 | 25 | 0.74 |
| | Osteoporotic | 1.95 | 1.54 | 2.46 | | 1.87 | 1.63 | 2.14 | 25 | 0.53 |
| | | | | | | | | | | |
| Yes | Any | 1.71 | 1.27 | 2.31 | | 1.79 | 1.50 | 2.14 | 24 | 0.60 |
| | Hip | 1.75 | 1.14 | 2.69 | | 1.53 | 1.16 | 2.02 | 15 | 0.25 |
| | MOF | 1.70 | 1.22 | 2.36 | | 1.63 | 1.33 | 2.00 | 22 | 0.59 |
| | Osteoporotic | 1.68 | 1.23 | 2.31 | | 1.71 | 1.42 | 2.07 | 23 | 0.84 |

MOF, Major osteoporotic fracture

**Table B** Hazard ratio (HR) and 95% confidence interval (CI) of fracture at the sites indicated associated with a history of prior fracture in men and women according to race/ethnicity. HRs are adjusted for age and time since baseline.

| **Outcome fracture** | **Number of cohorts** | **HR** | **95% CI** | **HR** | **95% CI** | **p-value** |
|---|---|---|---|---|---|---|
| **Asian vs White** | | **White** | | **Asian** | | |
| Any | 5 | 1.77 | 1.51-2.09 | 1.73 | 1.29-2.32 | 0.84 |
| Hip | 3 | 1.64 | 1.45-1.85 | 1.97 | 0.86-4.51 | 0.66 |
| MOF | 5 | 1.77 | 1.52-2.06 | 1.79 | 1.12-2.86 | 0.95 |
| **Black vs White** | | **White** | | **Black** | | |
| Any | 6 | 1.71 | 1.47-2.00 | 1.90 | 1.45-2.49 | 0.38 |
| Hip | 4 | 1.60 | 1.42-1.80 | 2.10 | 1.38-3.20 | 0.21 |
| MOF | 4 | 1.57 | 1.33-1.86 | 2.14 | 1.55-2.96 | 0.038 |
| **Hispanic vs White** | | **White** | | **Hispanic** | | |
| Any | 2 | 1.47 | 1.39-1.56 | 1.29 | 0.84-1.98 | 0.55 |
| Hip | 2 | 1.53 | 1.39-1.67 | 1.96 | 0.84-4.58 | 0.56 |
| MOF | 2 | 1.49 | 1.40-1.60 | 1.72 | 1.05-2.82 | 0.57 |
| **Other than White vs White** | | **White** | | **Other than White** | | |
| Any | 7 | 1.70 | 1.48-1.95 | 1.87 | 1.54-2.26 | 0.18 |
| Hip | 6 | 1.71 | 1.48-1.97 | 2.09 | 1.51-2.89 | 0.19 |
| MOF | 7 | 1.70 | 1.50-1.93 | 2.10 | 1.64-2.69 | 0.057 |

**Table C** Hazard ratio (HR) and 95% confidence interval (CI) of fracture at the sites indicated associated with a history of prior fracture in men and women combined according to quality score. HRs are adjusted for age and time since baseline.

| | Outcome fracture | Number of cohorts | HR | 95% CI | p[a] |
|---|---|---|---|---|---|
| High quality | | | | | |
| | Any | 27 | 1.88 | 1.62-2.19 | |
| | Hip | 26 | 1.71 | 1.44-2.03 | |
| | MOF | 25 | 1.84 | 1.53-2.23 | |
| | Osteoporotic | 26 | 1.87 | 1.60-2.19 | |
| Moderate quality | | | | | |
| | Any | 31 | 1.81 | 1.67-1.95 | 0.66 |
| | Hip | 29 | 1.82 | 1.64-2.01 | 0.54 |
| | MOF | 29 | 1.71 | 1.59-1.85 | 0.89 |
| | Osteoporotic | 28 | 1.78 | 1.65-1.92 | 0.58 |
| Low quality | | | | | |
| | Any | 4 | 2.00 | 1.23-3.26 | 0.81 |
| | Hip | 1 | 1.36 | 0.86-2.14 | 0.36 |
| | MOF | 1 | 5.47 | 2.05-14.55 | 0.033 |
| | Osteoporotic | 2 | 2.63 | 0.75-9.16 | 0.60 |
| [a] Two-sided p-values compared with high quality | | | | | |
